# Vitrified canine testicular cells allow the formation of spermatogonial stem cells and seminiferous tubules following their xenotransplantation into nude mice

**DOI:** 10.1038/srep21919

**Published:** 2016-02-24

**Authors:** Kyung Hoon Lee, Won Young Lee, Dong Hoon Kim, Seung Hoon Lee, Jung Tae Do, Chankyu Park, Jae Hwan Kim, Young Suk Choi, Hyuk Song

**Affiliations:** 1 Department of Animal Biotechnology, Konkuk University, 1 Hwayang-dong, Gwangjin-gu, Seoul 143-701, Republic of Korea; 2 Department of Food Bioscience, RIBH, College of Biomedical & Health Science, Konkuk University, Chung-ju 380-701, Republic of Korea; 3 Animal Biotechnology Division, National Institute of Animal Science, RDA, Wanju-gun 565-851, Republic of Korea; 4 Department of Biomedical Science, College of Life Science, CHA University, Seongnam 463-836, Korea

## Abstract

Belgian Malinois (BM), one of the excellent military dog breeds in South Korea, is usually castrated before sexual maturation. Therefore, the transfer of their genetic features to the next generation is difficult. To overcome this, testicular cells from 4-month-old BMs were frozen. Testicular cells were thawed after 3 months and cultured in StemPro-34 medium. Spermatogonial stem cell (SSC) characteristics were determined by the transplantation of the cultured germ cell-derived colonies (GDCs) into empty testes, containing only several endogenous SSCs and Sertoli cells, of immunodeficient mice, 4 weeks after busulfan treatment. Following the implantation, the transplanted cells localized in the basement membrane of the seminiferous tubules, and ultimately colonized the recipient testes. Xenotransplantation of GDCs together with testicular somatic cells conjugated with extracellular matrix (ECM), led to the formation of *de novo* seminiferous tubules. These seminiferous tubules were mostly composed of Sertoli cells. Some germ cells were localized in the basement membrane of seminiferous tubules. This study revealed that BM-derived SSCs, obtained from the castrated testes, might be a valuable tool for the transfer of BM genetic features to the next generation.

Male germ cell cultures have been previously established in mammals^1^,^2^. A culture of male germline stem cells from rodents has been maintained in mice and hamsters for 1 year and 5 months, respectively^3^,^4^. It was shown that human stage-specific embryonic antigen-4-positive spermatogonial stem cells (SSCs) can be cultured for 4 months without feeder cells^5^. Two types of media, StemPro-34 and Dulbecco’s Modified Eagle Medium (DMEM) supplemented with foetal bovine serum (FBS), have been used for SSC cultures derived from domestic animals. Colony formation has been observed in goat and pig SSC cultures grown in DMEM-FBS medium, and these colonies contained PGP9.5-positive cells^6^,^7^, which is regarded as a spermatogonia marker for domestic animals. In bovine, glial cell line-derived neurotrophic factor is important for the self-renewal and survival of SSCs, and plays a role in the proliferation of the cultured spermatogonial cells^8^. It was demonstrated previously that in SSC cultures derived from pigs, EGF and FGF have a positive effect on the number and size of SSC-like colonies, and the addition of EGF and FGF to the primary cell cultures of neonatal pig testes affects the expression of NANOG, PLZF, OCT4, and GATA4^9^. Furthermore, porcine germ cell-derived colonies (GDCs) were effectively formed at 31 °C in StemPro-34 medium, and the transplanted GDCs colonized the recipient testes 8 weeks post-transplantation. GFRα-1-positive germ cells exhibited the characteristics of SSCs^10^,^11^.

Cryopreservation is important for the maintenance of germ cells. Cryoprotective agents are effective for the cryopreservation of murine SSCs, and it was demonstrated that combining polyethylene glycol (PEG), dimethyl sulfoxide (DMSO), and FBS with murine SSCs, substantially improves germ cell recovery rate^12^. Supplementation of the medium with sugar molecules increased mouse SSC viability after thawing^13^.

*In vivo* transplantation of male germ cells has provided the evidence of SSC existence. These cells are recognized by their functional ability to reform spermatogenesis following transplantation and colonization in recipient rodent testes^2^,^11^,^14^,^15^. Xenografts of immature (neonatal or prepubescent) testicular cells are able complete spermatogenesis in the dorsal skin of immunodeficient mice^16^.Testis tissues that retain their normal functions, including normal spermatogenesis and formation of seminiferous tubules, have been observed in the xenografts of the isolated testicular cells, and it was shown that they can produce fertile sperm^17^,^18^.

Previously, we successfully established spermatogonial GDCs from 2-month-old beagle testes, which contain an abundance of undifferentiated testicular germ cells, and FGF was determined to be an important factor for the proliferation and colony formation of GDCs^19^.

However, a suitable method for the long-term preservation of castrated canine male germ cells has not been established thus far. The objective of this study was to identify the optimal conditions enabling the freezing of canine testicular cells for GDC culture, and to determine the SSC capacity of these GDCs. Here, we report the cryopreservation conditions for canine spermatogonial germ cells, and demonstrate their ability to form GDCs after thawing. Additionally, the GDCs established following the cryopreservation show SSC capacity and *de novo* testicular tissue formation in immunodeficient mice.

## Results

### Culturing and characterization of GDCs originating from BM germ cells

**Figure 1:**
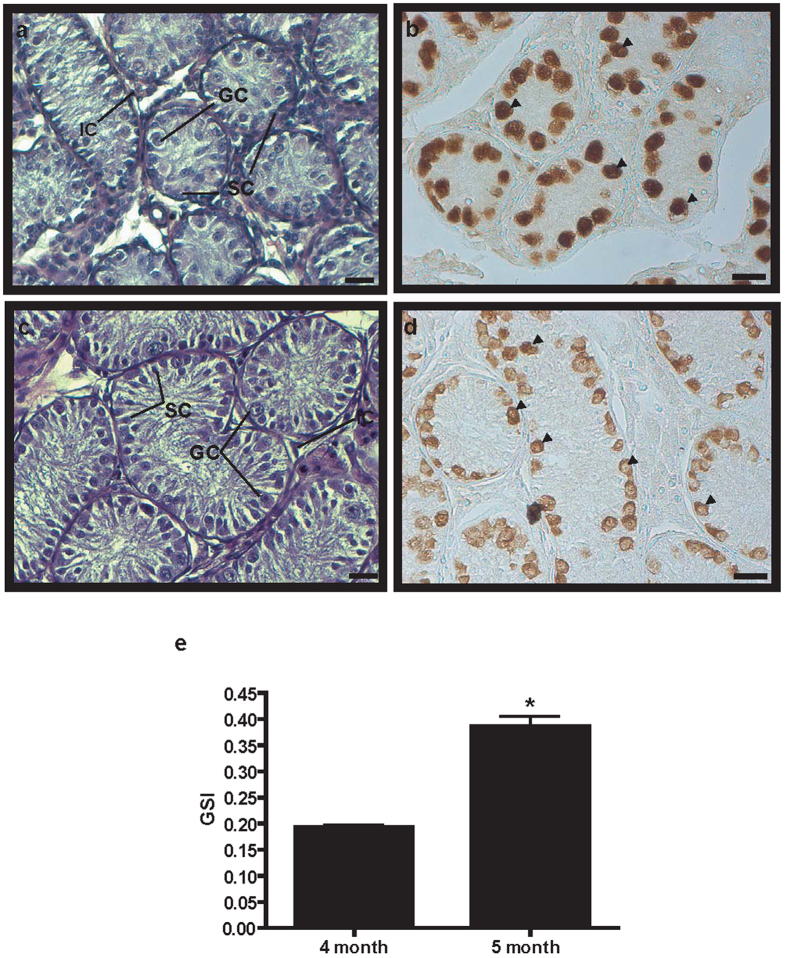
Histological and immunohistochemical analyses of 4- and 5-month-old BM testes. Panels (**a**,**c**) represent hematoxylin and eosin-stained testis sections in 4- and 5-month-old BMs. Testis sections of 4- (**b**) and 5-month-old (**d**) BMs were stained with anti-PGP9.5 antibody. Black arrowheads indicate PGP9.5-positive germ cells. Scale bars are 20 μm in all panels. GC, germ cell; IC, interstitial cell; SC, Sertoli cell. Magnification is 400× in all panels. Gonadal somatic index (GSI) = [Gonad Weight/Total Tissue Weight] × 100 (**e**). (*P < 0.05).

Histological analysis of the donated BM testes was performed, and testicular germ and Sertoli cells were observed in the seminiferous tubules of testes originating from both 4- and 5-month-old BMs, (Fig. 1a,c, respectively). The size of seminiferous tubule in 4-month-old BM testis was smaller than in 5-month-old BM testis. PGP9.5 protein-positive spermatogonial germ cells were detected in both 4- and 5-month-old BM testes, and aligned germ cells were located in the basement membrane of the seminiferous tubules (Fig. 1b,d). Gonado-somatic index of 5-month-old BM was significantly higher than that of 4-month-old BM was (Fig. 1e).

**Figure 2:**
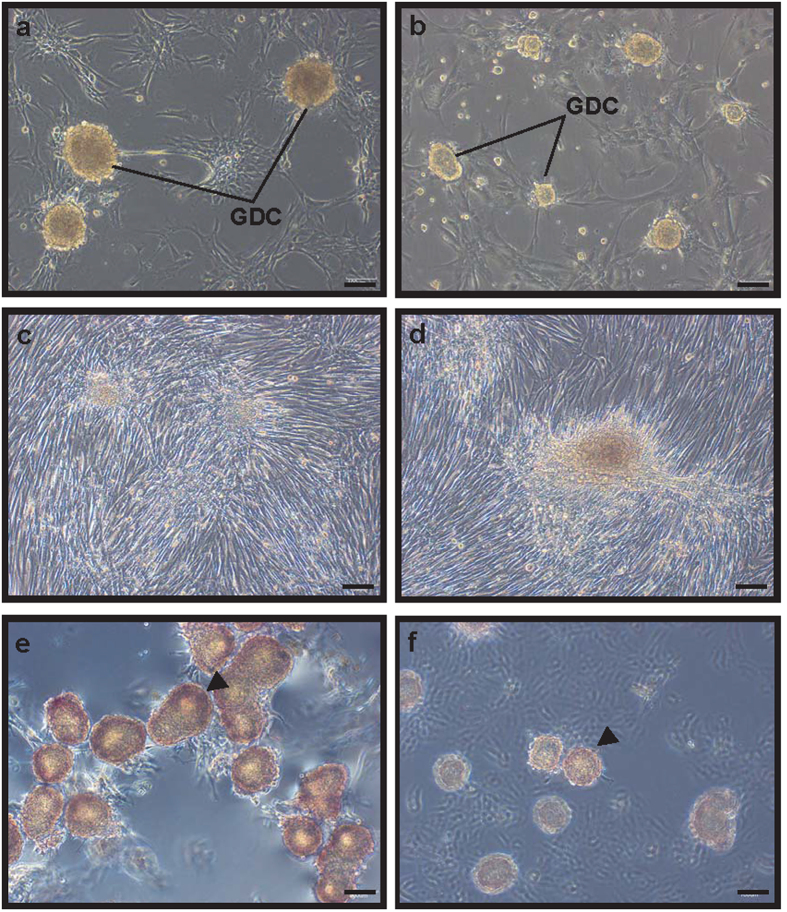
*In vitro* culture of primary testicular cells derived from 4- and 5-month-old BM testes. Testicular cells were cultured for 7 days in StemPro-34 (**a**,**b**) and DMEM-5% FBS (**c**,**d**). Panels a and c show testicular cells in 7-day culture derived from 4-month-old BM. Panels b and d show the images of the cells on the seventh day of the 5-month-old BM testicular cells culture. AP staining of GDCs in the culture of 4- (**e**) and 5- (**f**) month-old BM testicular cells. Scale bars are 100 μm and magnification is 100×. GDC, germ cell-derived colony.

GDCs were observed in the primary testicular cell culture originating from 4- and 5-month-old BM testes in StemPro-34 medium, although the GDCs in culture from 5-month-old BM testes were smaller than those from the 4-month-old-derived culture (Fig. 2a,b). GDCs were also observed in the culture of testicular cells from 4- and 5-month-old BM testes grown in DMEM-5% FBS media; however; they exhibited morphologic abnormalities in comparison with the GDCs cultured in StemPro-34 media (Fig. 2c,d). The colonies derived from all BMs were positive for alkaline phosphatase (AP) staining, but the staining intensity of GDCs in 4-month-old BM testes was stronger compared with the 5-month-old (Fig. 2e,f). Based on these results, 4-month-old BM testes and StemPro-34 medium were chosen for further experiments.

**Figure 3:**
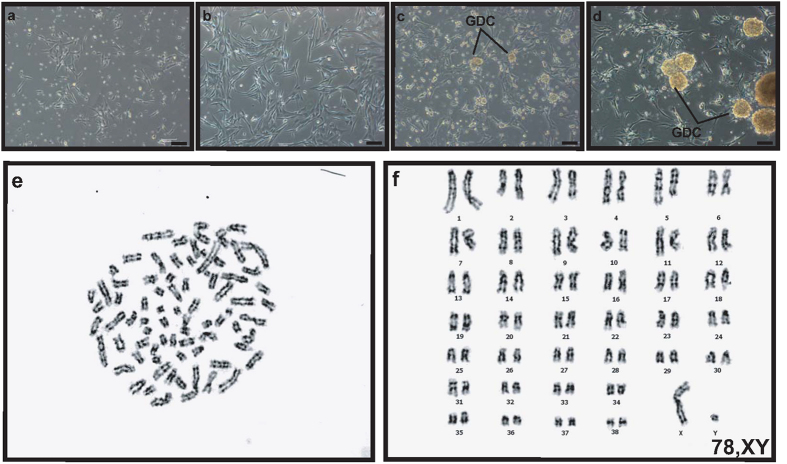
*In vitro* culture and karyotyping of cryopreserved testicular cells derived from 4-month-old BM testes after thawing. Total testicular cells were thawed following cryopreservation for 3 months. These cells, which were cryopreserved in StemPro-34 and DMSO (final concentration: 10%), were cultured for 7 days in StemPro-34 medium. Panels (**a**–**d**) show bright-field images captured at day 1, 3, 5, and 7, respectively. Panels e and f indicates G-banded metaphase spread in the cultures of primary and cryopreserved testicular cells, respectively. Scale bars are 100 μm and magnification is 100× in panels (**a**–**d**).

Using in-house-developed beagle testicular cell cryopreservation method (Supplementary Figs 1 and 2), testicular cells originating from 4-month-old BM testes were cryopreserved after a single suspension in StemPro-34 medium without cryoprotective agent (CPA). Three months after the cryopreservation, BM testicular cells were thawed and cultured in StemPro-34 medium. The recovery rate of the cryopreserved BM testicular cells from 4-month-old BM testes was 75.11 ± 3.05%. Cell adhesion to the bottom of the wells was noticeable already on the first day, and again observed on the third day (Fig. 3a,b). GDCs were first observed on the fifth day, and they continued to grow in size, which is shown in the images taken on the seventh day (Fig. 3c,d). Karyotype analyses of GDCs cultured from the cryopreserved testicular cells in StemPro-34 exhibited 78, XY normal chromosomes (Fig. 3e,f).

**Figure 4:**
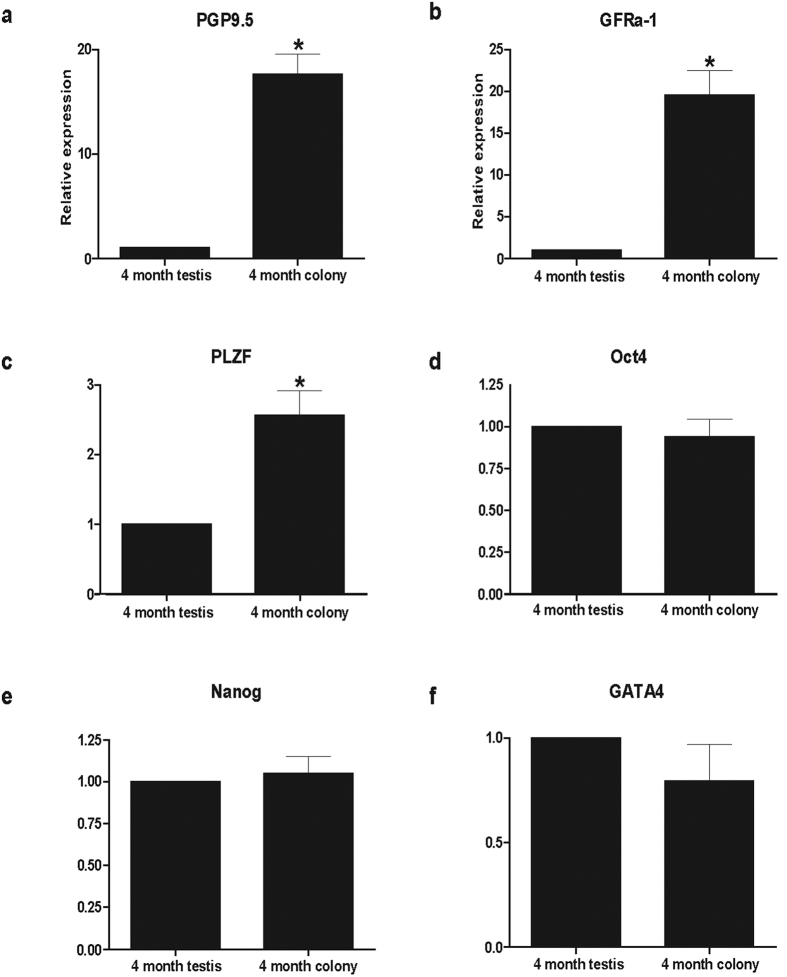
Real-time PCR gene expression comparison between BM testes and germ cell-derived colonies. Relative gene expressions were compared between GDCs and 4-month-old BM testes. *GAPDH*, glyceraldehyde 3-phosphate dehydrogenase; *PGP9.5*, protein gene product 9.5 (alternate designation: ubiquitin carboxy-terminal hydrolase L1); *GFRα-1*, glial cell line derived neurotrophic factor family receptor alpha 1; *PLZF*, promyelocytic leukaemia zinc finger; *Oct4*, octamer-binding protein 4; *Nanog*, homeobox transcription factor Nanog; *GATA4*, GATA-binding protein 4. (*P < 0.05).

The GDCs were collected on the seventh day, and real-time RT-PCR was performed, in order to identify the expression levels of stem cell and undifferentiated cell marker genes. As a result, *PGP9.5*, *GFRα-1*, and *PLZF* expression levels in GDCs were significantly (*P* < 0.05) higher than that in the 4-month-old BM testes were (Fig. 4a–c). No significant differences were observed in *Oct4*, *Nanog*, and *GATA4* expression levels between GDCs and 4-month-old BM testes (Fig. 4d–f).

### Xenotransplantation of GDCs into immunodeficient mice

**Figure 5:**
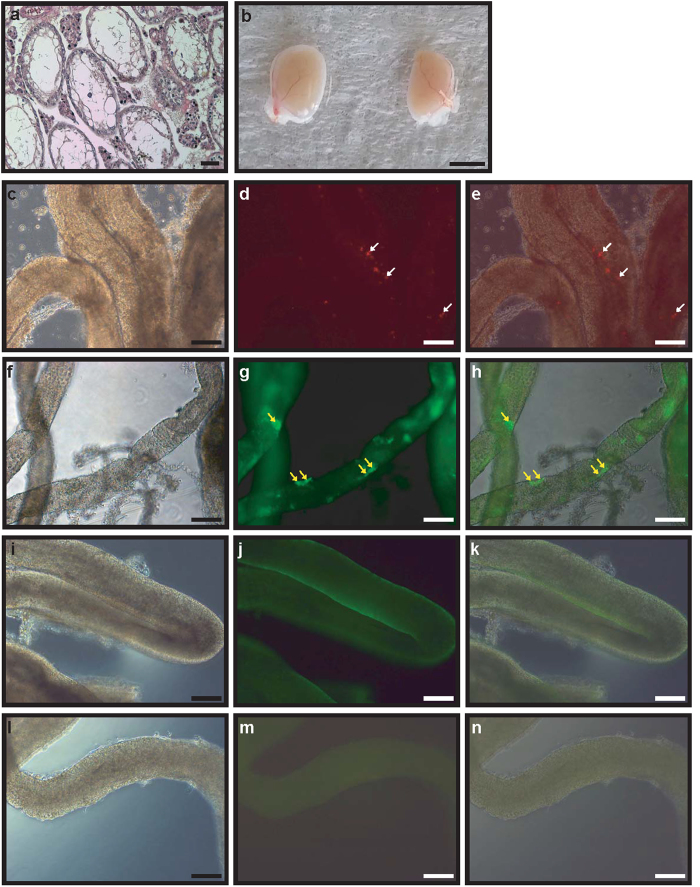
The detection of BM GDC cells after the transplantation of red fluorescent-labelled GDC cells into the seminiferous tubules of germ cell-depleted immunodeficient mice (12 weeks). Hematoxylin and eosin staining of busulfan-treated immunodeficient mouse testes (**a**). Red fluorescent-labelled GDC cells from 4-month-old BM testes (**b**, left testis) and GDC cells with no labelling (**b**, right testis) were transplanted into the immunodeficient mouse testes. A bright field image (**c**) and fluorescent image (**d**) of seminiferous tubules transplanted with red fluorescent-labelled GDC cells are shown. Panel (**e**) represents the merged image of panels (**c**,**d**). Panel (**f**) represents (**a**) bright field image, and panel g represents a fluorescent image of seminiferous tubules transplanted with red fluorescent-labelled GDC cells stained with anti-PGP9.5 antibody. Panel (**h**) is (**a**) merged image of panels (**f**,**g**). Panels (**i**,**j**) represent a bright field image (**i**) and a fluorescent image (**j**), respectively, of seminiferous tubules stained with anti-PGP9.5 antibody in a control mouse testis. Panels (**l**,**m**) show a bright field image (**l**) and fluorescent image (**m**), respectively, of seminiferous tubules without anti-PGP9.5 antibody staining in a control mouse testis. Panel (**k**) shows (**a**) merged image of panels (**i**,**j**) and panel (**n**) is (**a**) merged image of panels (**l**,**m**). White and yellow arrows indicate red fluorescent-labelled GDC cells and PGP9.5-positive cells, respectively. Scale bars are 50 mm in panel (**b**). Scale bars are 50 μm and magnification is 100× in panels a and (**c**–**n**).

Germ cell-depleted seminiferous tubules were observed in busulfan-treated mice 1 month after the treatment (Fig. 5a), and red fluorescent-labelled GDC cells were transplanted into them. No morphological differences were observed between non-GDC and GDC-injected testes (Fig. 5b). The examination of the seminiferous tubules of the injected mice by fluorescence microscopy showed that red-labelled cells were present in the seminiferous tubules (Fig. 5c–e). Whole mount immunohistochemical staining of red-labelled-GDC-injected mice detected PGP9.5-positive cells (male BM germ cells) in the seminiferous tubules, localized in their basement membranes (Fig. 5f–h). The seminiferous tubules of non-GDC-injected immunodeficient mice were stained with anti-PGP9.5 antibody, and PGP9.5-positive cells were not observed (Fig. 5i–k). PGP9.5-stained cells were not observed in the negative control samples either (Fig. 5l–n).

**Figure 6:**
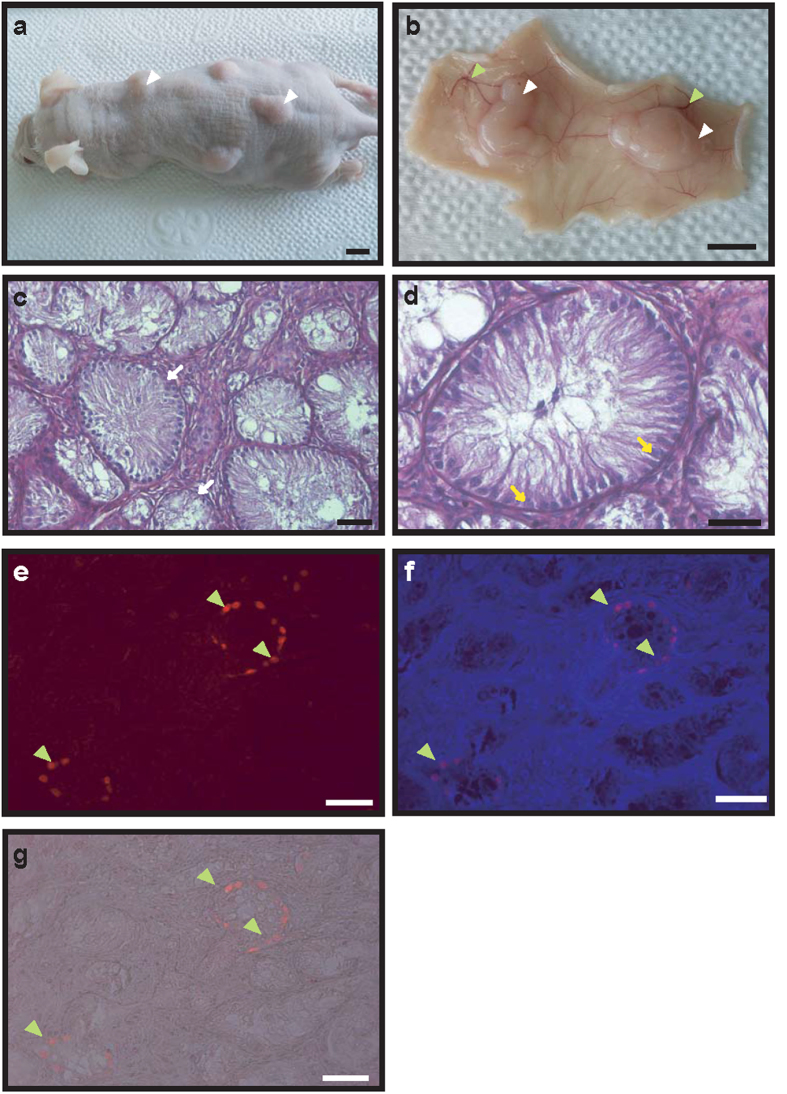
Neo-synthesis of BM seminiferous tubules following the xenotransplantation of GDC and testicular somatic cells into dorsal skin (20 weeks). Mouse xenografted with thawed GDC cells and testicular cells was sacrificed after 20 weeks (**a**). Panel (**b**) shows harvested tissues from the inner part of dorsal skin; white and green arrowheads indicate the tissues formed after xenotransplantation (GDC cells and testicular cells) and blood vessels, respectively. Scale bars are 50 mm in panels (**a**,**b**). Panel (**c**) shows hematoxylin and eosin staining of a tissue section in GDC cells and testicular cells xenograft at 20 weeks post-grafting, while panel (**d**) represents (**a**) magnified image; white and yellow arrows indicate seminiferous tubules and Sertoli cells, respectively. The tissue sections were stained with anti-PGP9.5 antibody (**e**), and panel (**f**) represents (**a**) merged image of panel e image and nuclear staining image. Panel g is a merged image of panel e and bright field image. Green arrowheads indicate PGP9.5-positive cells. Scale bars are 50 μm in all panels.

In order to determine the tissue regeneration capability of GDCs, the cryopreserved GDC cells were thawed and mixed with non-germ cells used as feeder layer for GDC culture, and matrigel, and afterward xenografted into the dorsal skin of immunodeficient mice. We observed tissue growth in these mice (Fig. 6a), and 20 weeks after the xenotransplantation, when the mice were sacrificed, the proliferated tissue was observed, with skin blood vessels connected with these newly grown tissues (Fig. 6b). The newly formed tissues were microscopically observed, and we noticed that blood vessels surrounded the tissues, and the putative seminiferous tubules could be seen clearly in the tissue sections (Fig. 6c). Additionally, no differentiating germ cells were observed in the tubules; only Sertoli-like cells were observed (Fig. 6d). To detect the germ cells in the seminiferous tubules, tissue sections were stained with anti-PGP9.5 antibody. Small populations of the PGP9.5-positive cells were observed, and they were shown to be located in the basement membrane of tubules (Fig. 6e–g).

## Discussion

Cryopreservation of mammalian SSCs using CPAs has been studied previously, and it was shown that cryopreservation with DMSO, 200 mM trehalose, and 2.5% PEG represents an efficient method in mice^12^,^13^,^20^. Additionally, undifferentiated male germ cells from pigs and bovine SSCs can be efficiently cryopreserved in the presence of 200 mM trehalose^21^,^22^. In our study, the highest recovery rate and colony number of the testicular cell cultures were shown for cells grown in non-CPA StemPro-34 medium; the use of CPAs resulted in a decreased recovery rate of cryopreserved testicular cells (Supplementary Figs 1 and 2). These data suggest that the type of freezing medium is more important than the choice of CPA for the cryopreservation of canine testicular cells.

Our previous studies showed that the testes of 2-month-old beagles had an abundance of undifferentiated germ cells, and that GDCs can be derived from these testes using StemPro-34 and DMEM supplemented with FBS media. Porcine GDCs can be cultured in StemPro-34 medium as well^11^,^19^. Here, we showed that BM GDCs cannot be formed in DMEM supplemented with 5 and 10% of FBS. However, StemPro-34 medium is sufficient for the culturing of GDCs derived from dogs and pigs. This indicates that StemPro-34 medium is widely acceptable basic medium for the derivation and proliferation of mammalian SSCs.

SSC transplantation into the seminiferous tubules of germ cell-depleted immunodeficient mice, in order to determine spermatogonial characteristics of domestic animals, has been reported. Pig-derived germ cells were able to colonize the seminiferous tubules of immunodeficient mice, but the subsequent stages of donor-derived spermatogenesis were not observed^23^. Our previous investigations demonstrated that the xenotransplantation of porcine GDCs to immunodeficient mice can lead to a successful colonization and localization of the GDCs at the seminiferous tubule basement membrane in the recipient testes^11^. Human SSCs survived in mouse testes for at least 6 months and proliferated during the first month after transplantation. No human differentiated spermatogonia were identified, and meiotic differentiation did not occur in mouse testes^24^, although the transplantation of mouse SSCs to immunodeficient mice resulted in the completion of spermatogenesis and successful offspring production^3^,^25^. Here, the seminiferous tubules of immunodeficient mice were colonized by GDC cells, but spermatogenesis was not completed; only the undifferentiated male germ cells were detected in the tubules. These results suggest that the microenvironment of mouse seminiferous tubules is not optimal for the domestic animal-derived germ cell transplantation.

Xenografting of mammalian immature testes into the dorsal skin of immunodeficient mice allows the completion of spermatogenesis *in vivo*^16^. However, the reports on xenotransplantation of *in-vitro* cultured or freeze-thawed testicular cells are rare. Functional testis tissues were observed 41 weeks following the implantation of isolated neonatal porcine testis cells under the skin of immunodeficient mice; somatic cells and germ cells reorganized into structures that were remarkably similar, both morphologically and physiologically, to normal testis tissue^26^. Additionally, complete peccary spermatogenesis, together with the production of fertile sperm, were observed in the tissues formed from testicular cell suspension xenografts 8 months post-grafting^18^. The presence of a basement membrane, a histologically normal interstitium, containing putative Leydig cells, the establishment of tubule lumen, and the integration of few putative spermatogonia into the seminiferous epithelium were observed in the xenografts of dissociated immature rat testicular cells^27^. Xenografting cells isolated from sheep testis tissues led to a complete spermatogenesis 40 weeks post-grafting^28^. In addition to rat testicular cells grafts, complete spermatogenesis has been achieved using cells originating from pig and sheep. We obtained reconstructed seminiferous tubules by the xenotransplantation of cultured BM GDCs and testicular somatic cells conjugated with ECM into the dorsal skin of immunodeficient mouse model. However, differentiated spermatogenic germ cells were not identified, and the majority of the observed cells were Sertoli cells; a few germ cells existed in the tubules. Similar morphology has been reported for dog-derived xenotransplants. Seminiferous tubules of cryptorchidic dogs are lined only by Sertoli cells, and these cells in atrophic tubules with impaired spermatogenesis often have large empty intracytoplasmic vacuoles^29^. In cryptorchidic mice, abdominal heat stress leads to the apoptosis of the testicular germ-cells^30^. Germ cell degeneration has also been observed in heat-treated pufferfish; *DMRT1*-expressing Sertoli cells represented the majority of cells in testicular tubules^31^. Furthermore, azoospermia was observed during suspension of the testicle to the skin at the scrotal neck for 1 year, but it was shown that short-term scrotal hyperthermia in dogs does not cause substantial changes in sperm quantity and quality^32^,^33^. Long-term heat treatment (>1 month) induces germ cell and spermatogenesis degeneration, and therefore, it is possible that the higher temperature (~36–37.5 °C) of nude mice caused germ cell depletion in the reconstructed BM testes following the xenotransplantation, because canine testis temperature is generally around 32–33 °C^33^,^34^,^35^.

Another possible explanation is that *in vitro* cultured BM GDCs are not fully functional in the reconstructed tubules, and that they are not capable of self-renewal and spermatogenesis, which leads to a possibility that the majority of the grafted BM GDC cells were degraded. Previous successful xenograft studies used freshly isolated cells from neonatal porcine testes^26^ and fragments of testis tissues, which contain functional spermatogonia and supporting physical microenvironment^18^,^28^. In support of this hypothesis, the xenografts of rat testicular cells that formed spherical aggregates on ECM coated dishes did not result in spermatogenesis, although seminiferous tubules were reconstituted^27^. Our research and previous studies suggest that the supporting environment plays an important role in germ cell differentiation.

In conclusion, StemPro-34 medium with DMSO was demonstrated to be optimal for the cryopreservation of canine testicular cells. Using this approach, germ cell characteristics are maintained in GDC culture after thawing. The transplanted BM GDC cells can colonize seminiferous tubules of the recipient mouse model. We showed that, following the xenotransplantation of these cells and testicular somatic cells with ECM into the dorsal skin of the recipient mice, very small population of germ cells is able to localize in the basement membrane of reconstituted seminiferous tubules. We conclude that the body temperature of recipient animals and the supporting GDC cell microenvironment should be taken into consideration during the xenotransplantation of GDC cells.

## Methods

### Testes preparation

Four beagles (2-month-old) were purchased from ORIENTBIO INC. (Seongnam, South Korea), castrated at Chungbuk National University, South Korea, and the obtained testes were used to establish cryopreservation conditions. Testes obtained from 4- and 5-month-old BMs were a donation from Korean Military Dog Training Camp (Chuncheon, South Korea). Gonado-somatic indices (GSIs) of BMs were calculated, and used for histology and immunohistochemistry, and testicular cell culture establishment. This study was carried out in strict accordance with the recommendation in the Guide for the Care and Use of Konkuk University Animal Care and Experimentation Community. The protocol was approved by the Committee on the Ethics of Animal Experiments of the Konkuk University (IACUC approval number: KU 15040).

### Cryopreservation, recovery rate analyses, and GDC number

Enzyme A (0.5 mg/ml collagenase, 0.01 mg/ml DNAse I, 0.1 mg/ml soybean trypsin inhibitor, and 0.1 mg/ml hyaluronidase) was added at 5-fold enzyme to tissue volume (v/w) to decapsulated testes, and incubated for 10 min at room temperature (RT). The testes were washed with PBS, and enzyme B (10 mg/ml collagenase, 0.01 mg/ml DNAse I, and 0.1 mg/ml soybean trypsin inhibitor) was added at a 5-fold volume of original testes weight (v/w), and incubated for 15 min at RT. Following this, the testes were washed with 10% Knockout Serum Replacement (Gibco, Carlsbad, CA, USA) in PBS, to inactivate the enzymes. These testes were meshed using a 40-μm nylon mesh, and red blood cells (RBCs) were eliminated using RBC lysis buffer (Sigma-Aldrich, St Louis, MO, USA). Two types of cell culture media were used for cryopreservation. StemPro-34 medium (Life Technologies, Carlsbad, CA, USA) was supplemented with insulin-transferrin-selenium (ITS; 25 μg/ml, 100 μg/ml, or 30 nM), 6 mg/ml glucose, 2 mM L-glutamine, 1% NEAA solution, 1% vitamin solution, 100 U/ml penicillin/streptomycin, 1 mM sodium pyruvate, 0.1 mM vitamin C, 1 μg/ml lactic acid, 30 ng/ml estradiol, 60 ng/ml progesterone, 0.2% BSA, and 1% Knockout Serum Replacement. DMEM (Life Technologies, Carlsbad, CA, USA) was supplemented with 10% FBS (DMEM-10% FBS). The isolated testicular cells of 2-month-old beagles were frozen in StemPro-34 medium with or without cryoprotective agents (CPAs: 100 mM or 200 mM trehalose, 2.5% PEG or 5% PEG), or in DMEM-10% FBS with or without CPAs (100 mM or 200 mM trehalose, 2.5% PEG or 5% PEG) for 3 months. DMSO (total concentration =10%) was added to all media. For each group, 1 × 10^6^ cells were added to a vial, together with 1 ml of a freezing medium. After 3 months, the cryopreserved beagle testicular cells were thawed at 37 °C for 1 min, and used to calculate recovery rates. The isolated testicular cells from 4- and 5-month-old BM testes were frozen in a StemPro-34-DMSO medium, as described previously for beagle testes. The cryopreserved BM testicular cells were thawed after 3 months, at 37 °C for 1 min and recovery rates were calculated. The formulas are as follows: recovery rate (%) = (number of recovered viable cells after freezing, thawing and washing/initial number of cells that were frozen, 1 × 10^6^ cells) × 100.

### Cell culture for GDC formation

The cryopreserved and primary testicular beagle cells were cultured in StemPro-34 medium in 12-well plate at 37 °C for 7 days, and colony morphology and cell karyotypes were compared with those of the cryopreserved testicular cells from the 4-month-old BM testes, which were cultured in StemPro-34 medium in 12-well plate at 37 °C for 7 days as well. Based on our previous reporoom, the cryopreserved and primary testicular cells from 4- and 5-month-old BM testes were cultured for 7 days in StemPro-34 medium, DMEM-5% FBS, and DMEM-5% FBS medium supplemented with 20 ng/ml mouse epidermal growth factor (mEGF, Millipore, Billerica, MA, USA, EA140), 10 ng/ml basic fibroblast growth factor (bFGF, Peprotech, Rocky Hill, NJ, USA, AF-100-18B), 10 ng/ml glial cell-derived neurotrophic factor (GDNF, R&D systems, Minneapolis, MN, USA, 512-GF-010), and 10^3 ^U/ml leukaemia inhibitory factor (LIF, Millipore, Billerica, MA, USA, EA140). GDC cells were collected and used for real-time PCR analyses, and frozen for xenografting.

### Karyotyping

The cryopreserved and primary testicular beagle and BM cells were incubated with 100 μl of colcemid solution (Irvine Scientific, Santa Ana, CA, USA, 9311) in StemPro-34 medium for 3 h at 37 °C, and the cells were treated with 1% citrate. Following the incubation and treatment, the cells were lysed and fixed in a methanol:glacial acetic acid (3:1) solution. G-band formation was analysed on each chromosome.

### Transplantation of BM GDC cells into busulfan-treated immunodeficient mouse testes

Busulfan (Sigma-Aldrich, St Louis, MO, USA, B2635; 40 mg/kg) was used in order to eliminate endogenous testicular germ cells in 4-week-old recipient BALB/c nude mice (n = 4) (ORIENTBIO INC., Seongnam, South Korea). GDC cells formed from cryopreserved testicular cell culture were isolated and labelled with 2 μM PKH26 Red Fluorescent Membrane Linker Dye (Sigma-Aldrich, St Louis, MO, USA, P9691) for 5 min. Afterward, PKH26-stained GDC cells were washed 3 times with DMEM and resuspended in the same medium containing 10% FBS. Trypan blue (0.001%) was used as an indicator of the injection into recipient seminiferous tubules. An aliquot of 1 × 10^5 ^cells/10 μl PKH26-labelled donor cells was injected into each recipient testis, which resulted in up to 80% filling of the recipient seminiferous tubules. Recipient testes injected with PKH26-labeled GDC cells were collected 8 weeks after the transplantation, and PKH26-positive cells and the localization of GDC cells in seminiferous tubules were detected by fluorescence microscopy, using an excitation filter of 450–560 nm (Nikon, Tokyo, Japan). Additionally, immunohistochemical analyses were performed, and seminiferous tubules were stained with anti-PGP9.5 antibody.

### Xenografting of BM GDC cells into the dorsal skin of immunodeficient mice

Single cells were separated from GDCs with 0.25% trypsin. Dorsal skin of 3 immunodeficient mice was injected with 5 × 10^5^ GDC cells, combined with 4.5 × 10^6^ feeder somatic BM testicular cells thawed from the cryopreserved cells (GDC cells:BM testicular cells =1:9), 50 μl StemPro-34 medium and 50 μl matrigel (BD Bioscience, Franklin Lakes, NJ, USA), using 23 gauge needle and 1-ml syringe. Immunodeficient mice xenografted with GDC cells and testicular somatic cells were sacrificed 20 weeks after the procedure and the tissues formed by GDC cells were collected. These tissues were observed under a microscope (Nikon, Tokyo, Japan), and afterward used for histology and immunohistochemistry.

### Histology and immunohistochemistry

Six-micrometre-thick slide sections of 4- and 5-month-old BM testes, busulfan-treated immunodeficient mice testes, and xenograft tissues were deparaffinized with xylene and treated with 100 to 50% ethanol. The sections were stained with hematoxylin and eosin and examined under a microscope (Nikon, Tokyo, Japan). For immunohistochemical analyses, the slides were incubated with a target unmasking fluid (Accurate Chemical & Scientific Corporation, Westbury, NY, USA) for 15 min, using a microwave oven to retrieve the antigens. The slides were washed thrice with PBS and blocked with 10% normal goat serum (v/v). For each PGP9.5 staining, a section of 4- or 5-month-old BM testis was incubated with anti-PGP 9.5 antibody (1:100; AbD Serotec, Raleigh, NC, USA), at 4 °C overnight, and then washed 3 times with PBS. Negative control slides were incubated with 1% BSA. Subsequently, slides were incubated with horse radish peroxidase-conjugated secondary antibody (1:500; Santa Cruz Biotechnology, Dallas, TX, USA) for 1 h, at RT (25 °C), followed by incubation in 3,3′-diaminobenzidine (Vector Laboratories, Burlingame, CA, USA). The slides were washed with PBS and then observed under a light microscope (Nikon, Tokyo, Japan). To detect PGP9.5, the tissue slides originating from xenografts were stained with anti-PGP 9.5 antibody (1:100; AbD Serotec, Raleigh, NC, USA) at 4 °C overnight, and then washed 3 times with PBS. As previously described, negative controls were incubated with 1% BSA. These slides were incubated with anti-mouse Alexa 568 and anti-rabbit Alexa 488 antibodies (both 1:500; Invitrogen, Carlsbad, CA, USA) against PGP 9.5, respectively, for 1 h at 25 °C (room temperature), followed by incubation with DAPI (Vector Laboratories, San Francisco, CA, USA). Afterward, the slides were washed with PBS and observed using an excitation filter of 450–560 nm and 200× magnification, under a fluorescence microscope (Nikon, Tokyo, Japan).

### Whole mount immunohistochemistry

Seminiferous tubules collected from BM GDC cell-transplanted immunodeficient mouse testes were fixed in 4% PFA at 4 °C, overnight. Following the fixation, they were washed 3 times with PBS and treated with 5% serum replacement (Gibco, Carlsbad, CA, USA) and 0.1% Triton X-100 for blocking. Seminiferous tubules were incubated with anti-PGP 9.5 antibody (1:100; AbD Serotec, Raleigh, NC, USA) at 4 °C overnight. Seminiferous tubules without BM GDC cell transplantation were used as controls. Negative control seminiferous tubules were incubated with 1% BSA. Afterward, all seminiferous tubules were incubated with anti-rabbit Alexa 488 (1:500; Invitrogen, Carlsbad, CA, USA) against PGP 9.5 antibody. This was followed by staining with 3,3′-diaminobenzidine (Vector Laboratories, Burlingame, CA, USA). The samples were washed with PBS and observed under a fluorescence microscope (Nikon, Tokyo, Japan) at 100× magnification.

### Real-time PCR

Total RNA from the cultured GDCs, originating from 4- or 5-month-old BM testes, was isolated using the RNeasy Mini Kit (Qiagen, Venlo, the Netherlands). cDNA templates were prepared from 1000 ng total RNA using Maxime RT Premix kit (iNtRON Biotechnology, INC., Seongnam, Korea). cDNA synthesis conditions were as follows: each cycle for 60 min at 94 °C, while the inactivation was performed for 5 min at 95 °C. Gene-specific primers used for beagle sample analysis were applied in the experiments with BM GDCs and testes as well^19^. Real-time PCR was performed with glyceraldehyde 3-phosphate dehydrogenase (*GAPDH*), *GFRα-1*, *PLZF*, *Oct4*, *Nanog*, and GATA-binding protein 4 (*GATA4*) primers, using Rotor-Gene Q (Qiagen, Venlo, the Netherlands). Real-time PCR conditions were as follows: 40 cycles of 20 s at 94 °C, 20 s at 60–62 °C, and 20 s at 72 °C, for all genes.

### Statistical analyses

Statistical analyses of recovery rate and colony number were performed using one-way nested analysis of variance (ANOVA) with Tukey test. Gonado-somatic indices and real-time PCR results were analysed using unpaired t-test with Welch’s correction analysis of variance. All analyses were performed using GraphPad Prism 4. Quantitative gene expression differences between colonies and testis tissue were calculated with Ct and delta-delta-Ct obtained in comparison with the GAPDH Ct value. The null hypothesis was rejected when the *P*-value was <0.05.

## Additional Information

**How to cite this article**: Lee, K. H. *et al.* Vitrified canine testicular cells allow the formation of spermatogonial stem cells and seminiferous tubules following their xenotransplantation into nude mice. *Sci. Rep.* **6**, 21919; doi: 10.1038/srep21919 (2016).

## Supplementary Material

Supplementary Information
