# Supplementary material for: Vitrified canine testicular cells allow the formation of spermatogonial stem cells and seminiferous tubules following their xenotransplantation into nude mice
Source: Sci Rep. 2016 Feb 24;6:21919. doi: 10.1038/srep21919 (PMC4764824; doi:10.1038/srep21919)
Supplement: Supplementary Information [file srep21919-s1.pdf]

Vitrified canine testicular cells allow the formation of spermatogonial stem cells and seminiferous tubules following their xenotransplantation into nude mice

Kyung Hoon Lee[1], Won Young Lee[2], Dong Hoon Kim[3], Seung Hoon Lee[3], Jung Tae Do[1], Chan Kyu park[1], Jae Hwan Kim[4], Young Suk Choi[4] and Hyuk Song[1]*

[1]Department of Animal Biotechnology, Konkuk University, 1 Hwayang-dong, Gwangjin-gu, Seoul 143-701, Republic of Korea

[2] Department of Food Bioscience, RIBH, College of Biomedical & Health Science, Konkuk University, Chung-ju 380-701, Republic of Korea

[3] Animal Biotechnology Division, National Institute of Animal Science, RDA, Wanju-gun 565-851, Republic of Korea

[4]Department of Biomedical Science, College of Life Science, CHA University, Seongnam 463-836, Korea

*Corresponding author: Hyuk Song (PhD), Department of Animal Biotechnology, College of Animal Bioscience and Technology, Konkuk University, Seoul 143-701, Korea

E-mail: songh@konkuk.ac.kr, Tel: +82-2-450-0562, Fax: +82-2-450-1044

Short title: Germ cell culture and xenotransplantation in BM

Supplementary figure legend

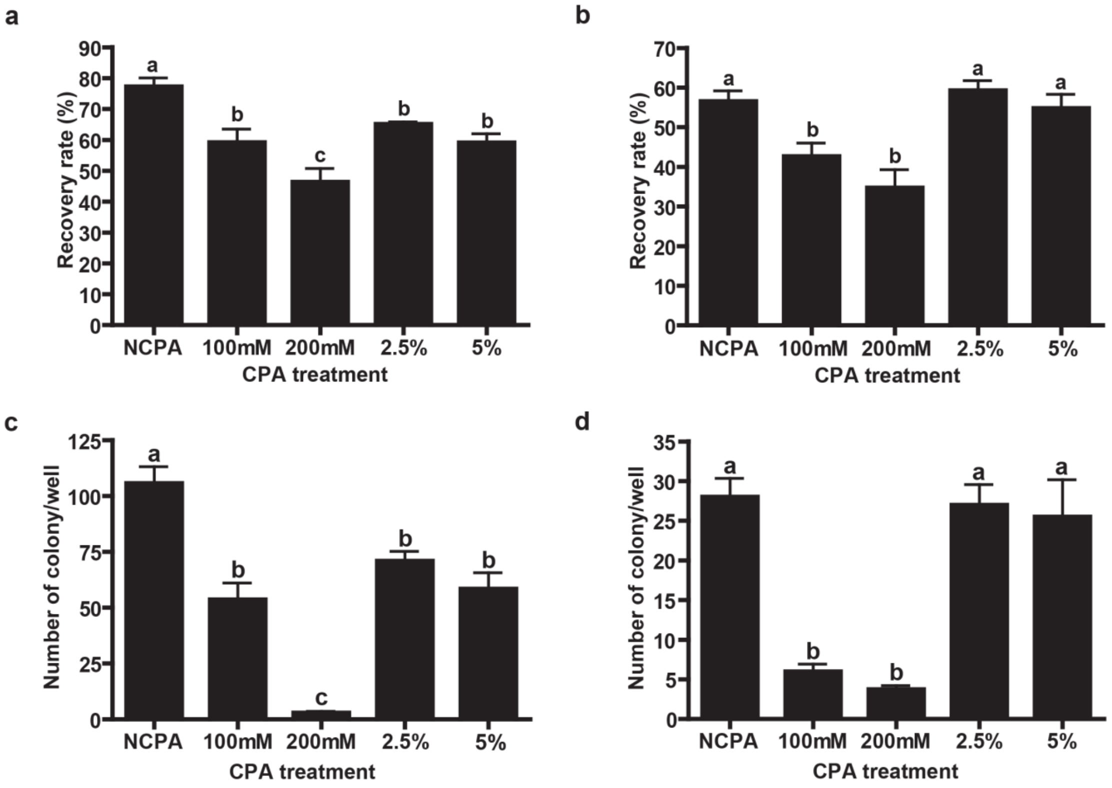

Supplementary Figure 1. Effect of cryoprotective agents on the recovery rate of cryopreserved testicular cells and the colony number in two-month-old beagle testis. Total testicular cells were thawed after cryopreservation for three months. Recovery rate of testicular cells cryopreserved in StemPro-34 (a) and DMEM-FBS (b) medium with DMSO (final concentration=10%) and different cryoprotective agents. The number of colonies in StemPro-34 medium after thawing from StemPro-34 (c) and DMEM-FBS (d) medium with DMSO and different cryoprotective agents. NCPA, non-cryoprotective agent group: 100mM, 100mM trehalose-added group; 200mM, 200mM trehalose-added group; 2.5%, 2.5% PEG-added group; 5%, 5% PEG-added group. $P<0.05$.

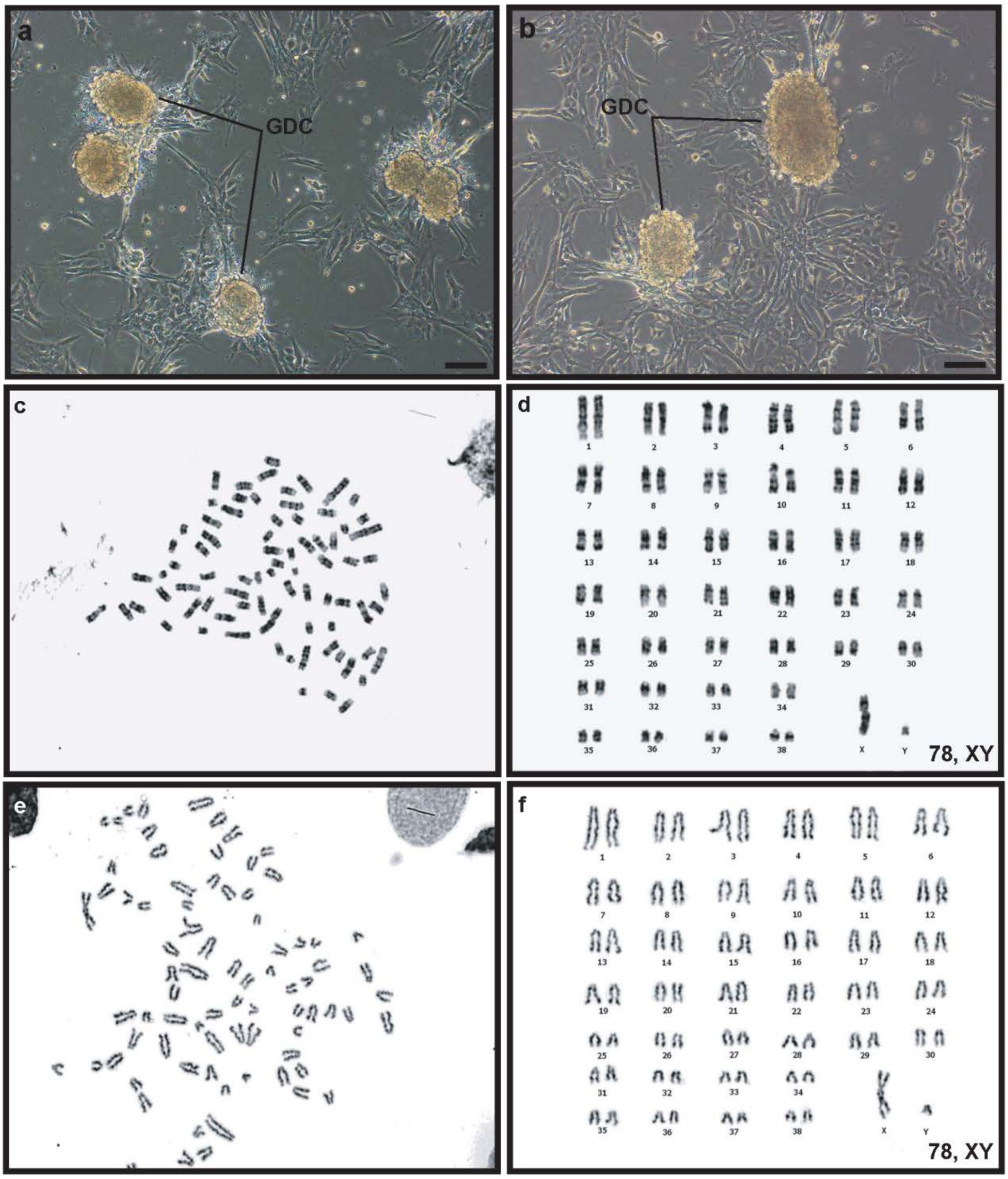

Supplementary Figure 2. Colony culturing using primary and cryopreserved testicular cells and karyotyping of cryopreserved two-month-old beagle testis testicular cells colony after thawing.

Morphology of primary cells GDCs in culture (a) and testicular cells previously cryopreserved in StemPro-34 and DMSO (b). Karyotypes of colonies from primary cultures (c and d) and cryopreserved (e and f) testicular cell cultures after thawing. Panel c and e indicate G-banded metaphase spread of the primary and cryopreserved

testicular cells culture, respectively. Scale bars are 100 μm and magnification is ×100 in panel A and B. GDC, germ cell-derived colony.
